# Morphological description of the tongue of the *Boa constrictor*

**DOI:** 10.1007/s11259-026-11248-7

**Published:** 2026-05-07

**Authors:** Thamara Cozzi Gonçalves, Isabella Bittencourt Pires Chaves, Rogério Antonio Ribeiro Rodrigues, Elane Guerreiro Giese, Érika Branco, Ana Rita de Lima

**Affiliations:** 1 Animal Health and Production, Amazon (PPGSPAA- UFRA), Belém, PA 66077-530 Brazil; 2 Institute of Animal Health and Production (ISPA), Federal Rural University of Amazonia (UFRA), Av. Presidente Tancredo Neves 2501, Belém, PA 66077-530 Brazil

**Keywords:** Tongue, *Boa constrictor*, Morphology, Snakes

## Abstract

The Amazon biome hosts a rich diversity of reptile species, including *Boa constrictor* (Linnaeus, 1758), widely distributed throughout Brazil and belonging to the order Squamata. Despite its ecological importance, detailed descriptions of lingual morphology in this species remain limited. This study aimed to describe the tongue of *B. constrictor* using macroscopic, histological, and scanning electron microscopy (SEM) analyses. Four specimens obtained post mortem after road accidents were analyzed. It is important to consider that freezing and thawing procedures may have influenced tissue preservation. Macroscopically, the tongue is elongated, bifurcated, and smooth, with no visible papillae or median groove. However, SEM revealed a median groove in the body of the tongue. Histologically, the tongue is lined by stratified squamous epithelium with slight keratinization, supported by connective tissue and skeletal muscle bundles. No papillae, taste buds, or salivary glands were observed under the conditions of this study. These findings support the sensory role of the tongue in snakes and contribute to comparative anatomical knowledge.

## Introduction

The Amazon biome harbors a high diversity of reptile species, including *Boa constrictor* (Linnaeus, 1758), a snake widely distributed in Brazil and belonging to the order Squamata (Medellín et al. 2000; Andrade et al. 2012). In Brazil, two subspecies are recognized: *Boa constrictor constrictor* and *Boa constrictor amarali* (Silva 2007).

Snakes rely on chemosensory mechanisms for environmental interaction, in which the tongue plays a fundamental role in transporting chemical particles to the vomeronasal organ (Schwenk 1995). Unlike mammals, in which the tongue participates in food manipulation, in snakes it is primarily associated with sensory function. In Boidae, the tongue is used to detect chemical and tactile stimuli involved in prey location (Scartozzoni and Molina 2004).

Macroscopically, the snakes tongue is typically elongated and bifurcated, with a smooth surface and absence of well-developed papillae. Microscopically, it is generally lined by stratified squamous epithelium, with varying degrees of keratinization, supported by connective tissue and skeletal muscle bundles. In many species, papillae and taste buds are absent or reduced, reinforcing the sensory role of this organ (Iwasaki and Kumakura 1994; El-Sayyad et al. 2011; Silva 2015). However, integrated analyses combining macroscopic, histological, and ultrastructural approaches in *Boa constrictor* remain scarce.

Therefore, this study aimed to describe the morphology of the tongue of *Boa constrictor* using complementary techniques, contributing to comparative anatomical knowledge.

## Materials and methods

Four specimens of *Boa constrictor* were used, which died after being run over, originating from the Paragominas Bauxite Mine - Paragominas- PA, under authorization SEMA-PA No. 455/2009 and 522/2009, cryopreserved and donated to the Animal Morphological Research Laboratory (LaPMA) of the Federal Rural University of Amazonia (UFRA).

The specimens were thawed in running water for approximately four hours and fixed with a 10% aqueous formaldehyde solution through intramuscular, subcutaneous, and intracavitary perfusion for conservation purposes. They were then kept submerged in tanks containing the same solution for a period of seven days. These procedures may have influenced tissue preservation and should be considered a limitation.

After fixation of the cadavers, the tongues were removed by means of a median incision of the skin and muscles of the ventral face of the mandible, in a longitudinal axis, connecting the mental symphysis to the cranial portion of the larynx, exposing the ventral face of the tongue. The lingual frenulum was sectioned, extending the incision to the caudal portion of the tongue, detaching it from the hyoid bone for effective extraction of the organ, followed by photo documentation and collection of fragments for histological evaluation at the Animal Histology and Embryology Laboratory (LHEA) of UFRA.

Macroscopic and histological analyses were performed on all four specimens. Scanning electron microscopy (SEM) was conducted on three specimens, as one specimen was reserved exclusively for gross anatomical evaluation. Therefore, all macroscopic and histological observations are based on four individuals, whereas ultrastructural findings are based on three samples.

The fragments of the *Boa constrictor* tongue were processed at UFRA’s LHEA using the routine histological protocol, being dehydrated in a growing series of alcohols (70% − 90%), diaphanized in xylene, soaked, and embedded in paraffin. The tissue sections were cut to a thickness of 5 μm to 6 μm using a Zeiss Hyrax 25 microtome and stained with hematoxylin and eosin (HE) and Gomori’s trichrome (light green) (Tolosa et al. 2003). The material was analyzed and photographed using a Leica DM 2500 photomicroscope, and the images were digitally captured (LAS CORE).

The samples of all fragments of interest from the tongue were also evaluated using scanning electron microscopy and processed according to Watanabe et al. (2007), being analyzed with a Tescan Vega 3 scanning electron microscope at the UFRA Electron Microscopy Laboratory.

For scanning electron microscopy processing, three tongues were fixed in 2.5% glutaraldehyde for 36 h, and then fragments were collected (one whole tongue was kept, and two fragments were removed from the root of the tongue). They were then washed three times in phosphate buffer. The material was then post-fixed in a 2% osmium tetroxide solution for 120 min. The samples were washed in phosphate buffer and then in distilled water. The samples underwent dehydration in ethyl alcohol at concentrations of 50% (1 h); 70% (1 h); 80% (1 h); 90% (1 h) and 100% (1 h). After dehydration with ethyl alcohol, the material was processed in a Balzers critical point device, CPD-030, using liquid CO2 for complete drying (Santana et al. 2013). The fragments were then mounted on aluminum metal bases (stubs) with carbon cement and covered with gold (Au) using the Lom Sputter Balzer SCD-040 device and analyzed using a Tescan Vega 3 scanning electron microscope (Gonçalves et al. 2022).

All nomenclature adopted was based on the International Committee on Veterinary Gross Anatomical Nomenclature (2017) and the International Committee on Veterinary Histological Nomenclature (2017).

## Results

### Macroscopy

**Fig. 1:**
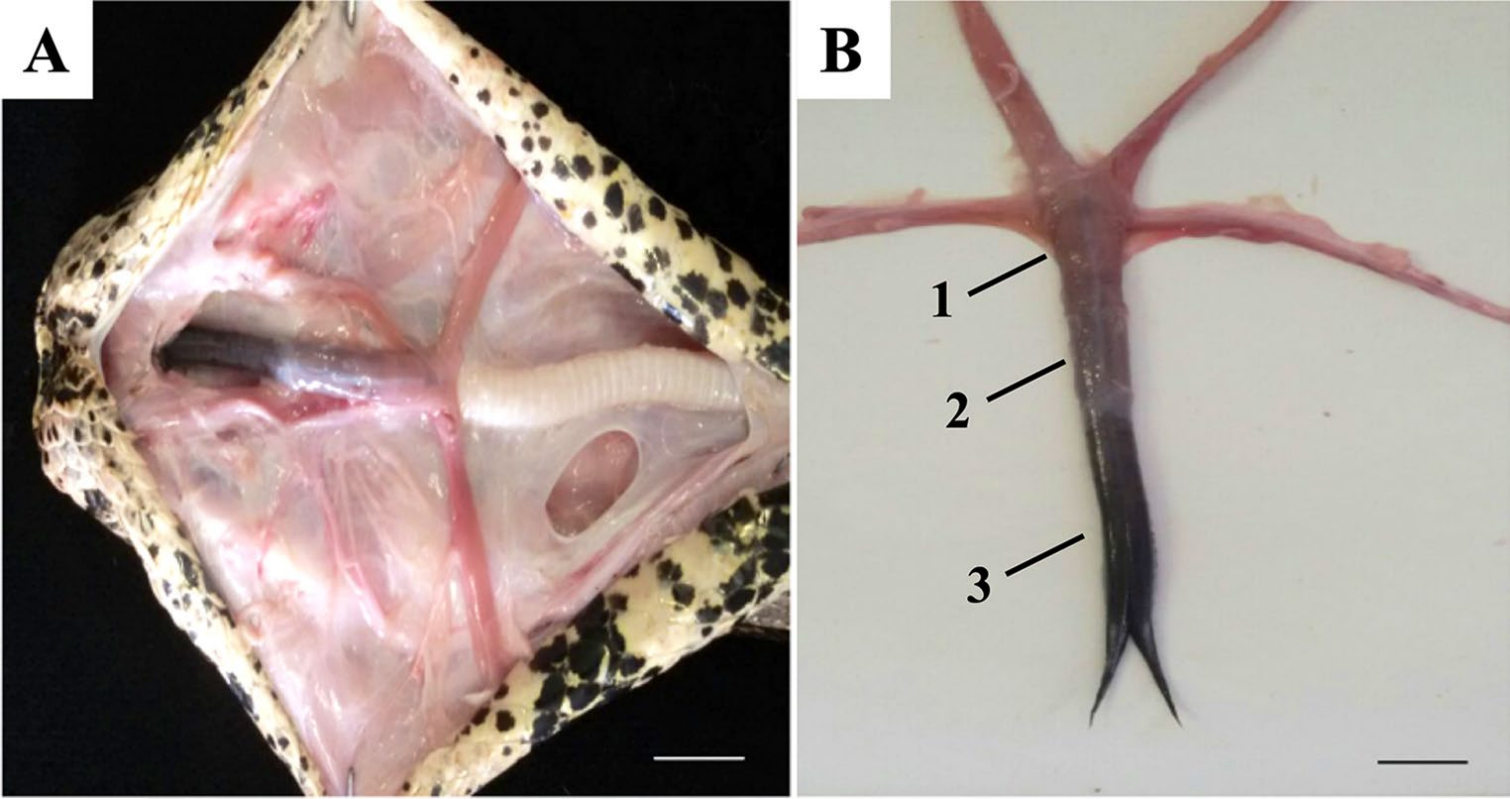
Dissection of the cephalic portion of *Boa constrictor* with exposure of the tongue. **A**- Opening of the oral cavity through a median incision exposing the ventral surface of the tongue (in situ). Scale bar: 1 cm. **B**- Total exposure of the dorsal surface of the tongue after removal. Root (1). Body (2). Apex (3). Scale bar: 1 cm

The tongue is elongated, narrow, and bifurcated, measuring approximately 6.2 cm. It consists of root, body, and apex, with smooth surface and dark coloration. No papillae or median groove were observed macroscopically (Fig. 1).

### Scanning electron microscopy (SEM)

**Fig. 2:**
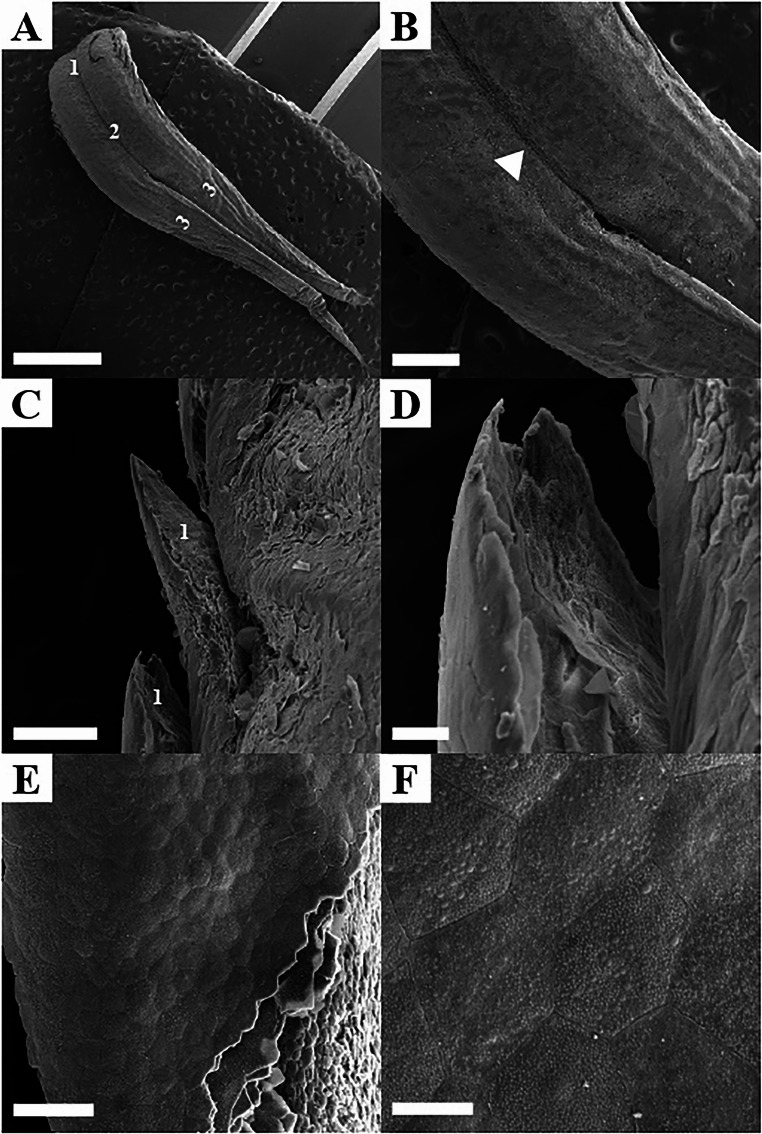
Scanning electron microscopy (SEM) of the tongue of *Boa constrictor*. **A**- Root of the tongue (1). Body of the tongue (2). Bifurcated tip of the tongue (3). Scale bar: 2 mm. **B**- Median groove (white arrowhead). Scale bar: 500 μm. **C**- Lateral projections at the root of the tongue (1). Scale bar: 100 μm. **D**- Lateral projections at higher magnification and absence of papillae on the tongue of *Boa constrictor*. Scale bar: 20 μm. **E**- Layered arrangement of keratinized stratified squamous epithelial tissue. Scale bar: 50 μm. **F**- Hexagonal block conformation of keratinized stratified squamous epithelial tissue. Scale bar: 10 μm

Scanning electron microscopy (SEM) revealed that the tongue of *Boa constrictor* was aglandular and divided into root, body, and bifurcated apex (Fig. 2A). A median groove was identified in the body region, although it was not visible macroscopically (Fig. 2B). No papillae were identified on the entire surface of the boa’s tongue, but there were lateral projections in the lingual root region (Fig. 2C and D). The outermost surface of the tongue showed a layered arrangement with a hexagonal block conformation of keratinized stratified squamous epithelial tissue (Fig. 2E and F).

### Light microscopy

**Fig. 3:**
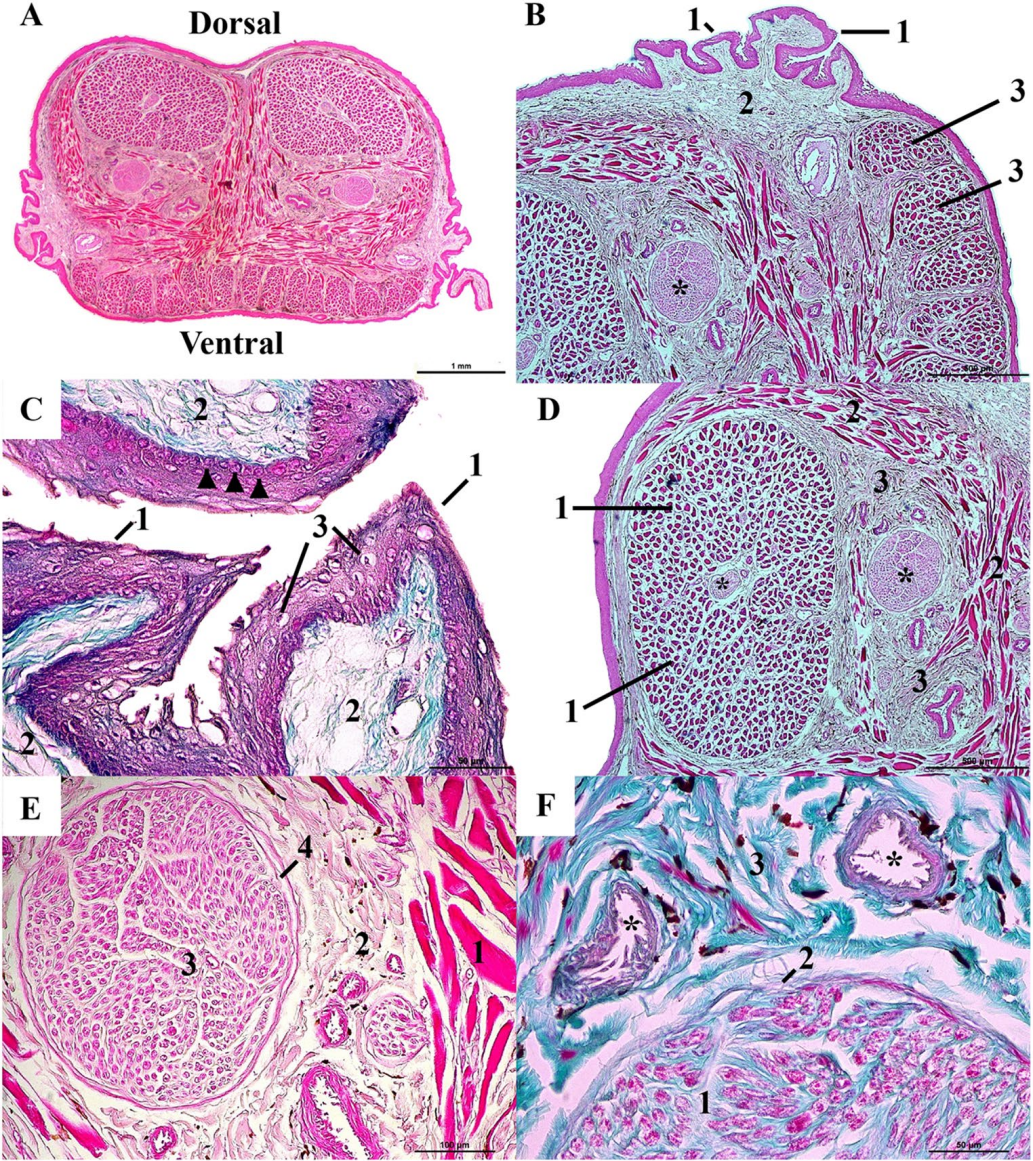
Light microscopy of the tongue of *Boa constrictor*. **A** - Cross-sections of the tongue body region. Dorsal and ventral portion. Scale bar: 1 mm. Staining: Hematoxylin-Eosin (HE). **B**- Transverse section of the root of the tongue. Lateral projections of tissue (1). Loose connective tissue (2). Transverse fibers of skeletal muscle tissue rearranged into trabeculae (3). Nerve (black asterisk). Scale bar: 500 μm. Staining: Hematoxylin and Eosin (HE). **C**- Stratified keratinized squamous epithelial tissue (1). Loose connective tissue (2). Flattened cells in the outer layer of the epithelium (3). Cubic cells in the basal layer of the epithelium (black arrowheads). Scale bar: 50 μm. Staining: Gomori’s trichrome (Light Green). **D**- Transverse fibers of skeletal muscle tissue rearranged into trabeculae (1). Longitudinal fibers of skeletal muscle tissue (2). Dense irregular connective tissue (3). Nerve (black asterisk). Scale bar: 500 μm. Staining: Hematoxylin and Eosin (HE). **E**- Longitudinal fibers of skeletal muscle tissue (1). Dense irregular connective tissue (2). Endoneurium (3). Perineurium (4). Scale bar: 100 μm. Staining: Hematoxylin and Eosin (HE). F- Endoneurium (1). Perineurium (2). Dense irregular connective tissue (3). Blood vessels (black asterisks). Scale bar: 50 μm. Staining: Gomori’s trichrome (Light Green)

Microscopically, cross-sections of the tongue body of *Boa constrictor* revealed distinct dorsal and ventral regions (Fig. 3A). The tongue is covered by discretely keratinized stratified squamous epithelium, supported by loose and dense irregular connective tissue (Fig. 3B and D). The basal layer consists of cuboidal germinative cells responsible for epithelial renewal, while the outer layer is composed of flattened cells (Fig. 3C).

No salivary glands, lingual papillae, or taste buds were observed; only lateral projections were present at the root region (Fig. 3B). Skeletal muscle fibers were arranged in transverse and longitudinal orientations, with a more robust layer near the ventral surface (Fig. 3D). Nerve segments, including endoneurium and perineurium regions, were identified within the connective tissue between muscle layers, associated with blood vessels (Fig. 3D, E and F).

## Discussion

According to Junqueira and Carneiro (2017), most carnivorous mammals have most of their oral cavity occupied by the tongue. Only the apex portion is free, with the rest of the organ attached to the floor, in addition to having a macroscopically evident median groove (Dyce et al. 2019). *Boa constrictor*, like other snakes, has a narrower tongue and does not occupy most of the oral cavity, as mammals do. Some species of lizards and snakes also have a clearly visible median groove (Silva 2015), such as those described by (El-Sayyad et al. 2011), which have a median groove, but it can only be observed through electron microscopy, as described in this study.

In mammals, the tongue plays an important role in food processing, exhibiting structural variations, including different types and distributions of papillae adapted to feeding functions (Scartozzoni and Molina 2004). In contrast, the tongue of snakes is primarily associated with sensory function. In *B. constrictor*, it is involved in tongue-flicking behavior, characterized by the projection and retraction of the tongue to collect chemical particles, which are then transferred to the vomeronasal organ for analysis (Schwenk 1995; Silva 2015).

The functional organization of the tongue in *Boa constrictor* is closely related to the regional arrangement of skeletal muscle fibers. The root region, dominated by longitudinal muscle bundles such as the *m. hyoglossus*, is primarily involved in protrusion, whereas the apex bifurcated region presents a more complex arrangement of longitudinal, transverse, and vertical fibers that allow rapid oscillatory movements. These findings indicate regional variation in muscle organization, reflecting distinct functional roles (Schwenk 1995; de Groot et al. 2004).

The absence of taste buds on the tongue of *B. constrictor* has been observed, however Schwenk (1995) reports the presence of scattered taste buds in the oral cavity of Squamata in general. According to Schwenk (1988), snakes have a thin layer of keratinized tissue or only cells that are components of stratified squamous epithelial tissue and no papillae. The tongue coating of *B. constrictor* differs from *Elaphe quadrivirgata* (Iwasaki and Kumakura 1994) and *Psammophis sibilans* (El-Sayyad et al. 2011), since that of *B. constrictor* showed slight keratinization, while the other species mentioned were reported to have no keratinization.

Some species of the Iguanidae family, such as *Oplurus cuvieri* (Deuheusy et al. 1994), also have a smooth, soft tongue with few papillae. Unlike *B. constrictor*, some species of the order Crocodylia, such as *Crocodylus niloticus* (El-Sayyad et al. 2011), have a highly keratinized tongue and thickened epithelium, and *Alligator mississippiensis* (Shimada et al. 1990) has filiform and fungiform papillae across the entire dorsal surface of the tongue.

The lateral projections found in *B. constrictor* are also reported by El-Sayyad et al. (2011), in which *Psammophis sibilans* has tissue projections, called microfacets, followed by regularly spaced pores.

Based on the microscopic characteristics observed in the tongue of *Boa constrictor* in this study, such as the absence of papillae, the presence of keratinization, and lateral projections, it is possible to infer similarities with descriptions previously reported in other snakes and lizards. However, confirmation of the absence of taste buds requires further studies with a larger sample size.

## Data Availability

No datasets were generated or analyzed during the current study.
